# 

**Published:** 1827-03

**Authors:** 


### MONTHLY LIST OF MEDICAL BOOKS.

[*No books can be entered on this List except those sent to us for the purpose; as, in the list hitherto transmitted, the names of works have frequently been given as published, which have not appeared for weeks, or even months, after.*]

Outlines of Human Physiology. By Herbert Mayo, Surgeon, and Lecturer on Anatomy.

Appendix to the Papers on the Nerves, republished from the Royal Society's Transactions, by Charles Bell: containing Consultations and Cases illustrative of the Facts announced in these Papers.

On the Treatment of the more Protracted Cases of Indigestion. By A. P. W. Philip, M.D. F.R.S. L. & E. Being an Appendix to his Treatise on Indigestion.

Observations on the Treatment of Gonorrhœa by a new Preparation from the Balsam of Copaiba; with illustrative Cases. By James Thorn, M.R.C.S.

An Introductory Lecture to a Course of Surgery, delivered at the Richmond School of Medicine, Dublin, Jan. 8th, 1827. By R. Carmichael, Esq. M.R.I.A.

A Critical Analysis of the Memoir read by Dr. Barry before the Academy of Sciences, on the 8th of June, 1825, at the Institute of France, on Atmospheric Pressure being the principal Cause of the Progression of the Blood in the Veins. By Henry Searle, Surgeon.

Observations on the Expediency of instituting a Friendly Association of the Medical Profession throughout Scotland, for insuring a Provision during Sickness and old Age, Widows' Annuities, Endowments to Children, &c. By E. D. Allison, Esq.

Proceedings at the Eighth Anniversary Meeting of the Hunterian Society.

Medical Botany, No. II.

### METEOROLOGICAL JOURNAL,

### NOTICES.

*Numerous Communications from Gentlemen resident in London have been privately acknowledged.*

*The Communications of* Dr. Heineken, Mr. Cox, Mr. Mackie, Mr. Smith, Mr. Allison, *and* Mr. Mackenzie, *have been received.*

*We are much obliged by, and shall avail ourselves of,* Dr. Forbes's *polite offer.*

